# ﻿*Dysosma xishuiensis* (Berberidaceae), a new species from Guizhou, China, based on morphological and molecular evidence

**DOI:** 10.3897/phytokeys.268.152287

**Published:** 2025-12-15

**Authors:** Lang Huang, Wei-Hao Yao, Yan-Bing Yang, Ming-Tai An, Yuan-Lin Yu, Mei Zhou, He Li

**Affiliations:** 1 Guizhou Academy of Forestry, Guiyang 550005, Guizhou, China Guizhou Academy of Forestry Guiyang China; 2 Key Laboratory for Biodiversity Conservation in Karst Mountain Area of Southwestern China, National Forestry and Grassland Administration, Guiyang 550005, Guizhou, China Key Laboratory for Biodiversity Conservation in Karst Mountain Area of Southwestern China, National Forestry and Grassland Administration Guiyang China; 3 College of Forestry, Guizhou University, Guiyang 550025, Guizhou, China Key Laboratory for Biodiversity Conservation in Karst Mountain Area of Southwestern China, National Forestry and Grassland Administrasstion Guiyang China; 4 Center for Biodiversity and Natural Conservation, Guizhou University, Guiyang 550025, Guizhou, China Guizhou University Guiyang China; 5 Guizhou Xishui National Nature Reserve Management Bureau, Xishui 564600, Guizhou, China Guizhou Xishui National Nature Reserve Management Bureau Xishui China

**Keywords:** *
Dysosma
*, molecular phylogeny, morphology, new taxon, taxonomy

## Abstract

*Dysosma xishuiensis* Y. B. Yang, M. T. An & C. H. Yang is described and illustrated as a new species from Xishui County in northern Guizhou Province, China. This species is morphologically similar to *D. versipellis*, but differs by its glabrous abaxial leaf surface, pedicels, and sepals; inflorescences bearing more flowers (8–20 vs. 5–8); larger obovate–oblong petals (2.8–3.4 × 1.5–2.0 cm vs. 2.5 × 0.8 cm); and an obpyriform ovary (vs. ellipsoid). The phylogenetic relationships reconstructed using ITS, *matK*, and *rbcL* sequences further confirm that it is a new species within *Dysosma*. This species is currently known only from the Xishui National Nature Reserve in Guizhou. According to IUCN criteria (B2, D), due to its limited distribution and low population numbers, *D. xishuiensis* is assessed as Endangered (EN).

## ﻿Introduction

*Dysosma* Woodson (Podophylloideae, Berberidaceae) is a small genus comprising nine species, primarily distributed in China and Vietnam. China is considered the center of diversity and origin for *Dysosma* (Ma and Hu 1997), with the eastern Yunnan–Guizhou Plateau to the Three Gorges region being a key center for the distribution and diversification of *Dysosma* species (Ying 1979). *Dysosma* is closely related to *Sinopodophyllum* (Royle) T.S. Ying, *Podophyllum* L., and *Diphylleia* Michx., all of which belong to the Podophylloideae subfamily, but *Dysosma* can be distinguished by its perennial growth habit, creeping rhizomes, fibrous roots, 3–9-lobed or peltate leaves, umbellate inflorescences, and berries containing numerous seeds (Stähelin and Von 1991; Ying et al. 2011). Except for *Podophyllum*, the other three genera are distributed in China. Recent phylogenetic studies have resolved the relationships among *Dysosma*, *Sinopodophyllum*, *Podophyllum*, and *Diphylleia*, supporting that *Dysosma* is sister to a clade comprising *Sinopodophyllum* and *Podophyllum* (Loconte and Estes 1989; Nickol 1995; Kim and Jansen 1998; Liu et al. 2002; Wang et al. 2007; Mao et al. 2014; Ye et al. 2018; Hsieh et al. 2022). Currently, nine species are recognized in *Dysosma*, including seven species described in the “Flora of China”. Additionally, *D. villosa* Z.W. Wang & H.C. Xi was recently described (Wang et al. 2019), and *D. tonkinense* (Gagnep.) M. Hiroe has been proposed as a distinct species (Pham et al. 2020). It is noteworthy that Shaw (2002) and Ying et al. (2011) indicated that three insufficiently studied taxa originally placed in *Podophyllum*, namely *P. glaucescens* J.M.H. Shaw, *P. hemsleyi* J.M.H. Shaw & Stearn, and *P. trilobulum* J.M.H. Shaw, may actually belong to *Dysosma*. Due to insufficient specimens for detailed study, the taxonomic status of these three taxa remains controversial. In medicine, plants of the genus *Dysosma* have been widely used as herbal remedies primarily for treating throat swelling and pain, venomous snake bites, bruises and sprains, lymph node inflammation, and related disorders (Woodson 1928; Ying et al. 2011; Mao et al. 2014; Pham et al. 2020).

During our field investigation in Xishui National Nature Reserve, Xishui County, Guizhou Province, China (November 2021 and May 2022), we found an unusual population of *Dysosma* species. Initially, the plant seemed similar to *D. versipellis* in its stems (erect, unbranched, glabrous), leaves (alternate, blade suborbicular, to 25–30 cm in diameter, palmately lobed), and flowers (attached near the base of the blade, red). However, further examination revealed several key diagnostic features that differed from those of *D. versipellis*, such as a glabrous abaxial leaf surface, pedicels, and sepals; inflorescences bearing more flowers (8–20 vs. 5–8); larger obovate–oblong petals (2.8–3.4 × 1.5–2.0 cm vs. 2.5 × 0.8 cm); and an obpyriform ovary (vs. ellipsoid). To determine the taxonomic status of this taxon, we conducted systematic molecular studies using nuclear ITS and plastid markers (*matK*, *rbcL*) and inferred its position within Berberidaceae. Both morphological and molecular evidence support that this distinct population represents a novel species; thus, we formally describe it here.

## ﻿Materials and methods

### ﻿Morphological studies

The morphological study of the new species was based on field observations of over 10 living individuals in Xishui National Nature Reserve. Comparison of morphological characteristics with four other closely related species was supplemented by high-resolution digitized herbarium specimens accessed via the Chinese Virtual Herbarium (**CVH**; https://www.cvh.ac.cn/) and JSTOR Global Plants (https://plants.jstor.org/). Detailed observations and measurements were performed to document key diagnostic features. Voucher specimens of the new species have been deposited in the Herbaria of the Forestry College, Guizhou University (**GZAC**); the Guizhou Academy of Forestry Science (**GF**); and the Kunming Institute of Botany, Chinese Academy of Sciences (**KUN**).

### ﻿Taxon sampling, DNA sequencing, and molecular analysis

We collected one individual from each of the two natural populations within the protected area and dried the fresh leaf samples using silica gel. Genomic DNA was extracted from the dried leaves using a modified CTAB protocol (Doyle and Doyle 1987). DNA sequencing, primer design, and PCR amplification followed protocols from Mao et al. (2014). We retrieved ITS, *matK*, and *rbcL* sequences of 13 species (totaling 39 sequences) from GenBank to resolve the phylogenetic position of the new species. These encompassed representatives from Podophylloideae (Berberidaceae): *Dysosma* (seven species), *Diphylleia* (three species), *Podophyllum* (one species), and *Sinopodophyllum* (one species), with *Achlys triphylla* (Berberidaceae) serving as the outgroup. Corresponding GenBank accession numbers are presented in Table 1.

**Table 1.** Species names and GenBank accession numbers of ITS, *matK*, and *rbcL* sequences used for analysis. Superscripts denote two distinct populations of the new species.

Species	ITS	*matK*	*rbcL*
*Achlys triphylla*	MG235275	MG593050	MG593050
*Diphylleia cymosa*	KC494675	KC539368	KC539405
*Diphylleia grayi*	KC494679	KC539373	KC539409
*Diphylleia sinensis*	KC494674	KC539367	KC539403
*Dysosma aurantiocaulis*	KC494665	KC539356	KC539395
*Dysosma delavayi*	KC494672	KC539365	KC539402
*Dysosma difformis*	KC494660	KC539359	KC539390
*Dysosma majoensis*	KC494662	KC539353	KC539392
*Dysosma pleiantha*	KC494652	KC539345	KC539382
*Dysosma tsayuensis*	KC494668	KC539361	KC539398
*Dysosma versipellis*	KC494658	KC539351	KC539388
***Dysosma xishuiensis ^1^***	**PX138794**	**PX229892**	**PX229894**
***Dysosma xishuiensis ^2^***	**PX138795**	**PX229893**	**PX229895**
*Podophyllum peltatum*	KC494685	KC539378	KC539415
*Sinopodophyllum hexandrum*	KC494684	KC539377	KC539413

All sequences were aligned using MAFFT v7.505 (Katoh and Standley 2013), trimmed with Gblocks 0.91b (Talavera and Castresana 2007), and concatenated to generate the final alignment matrix. Phylogenies were subsequently reconstructed using maximum likelihood (ML) and Bayesian inference (BI). ML analysis was implemented in IQ-TREE v1.6.12 (Nguyen et al. 2015) with the GTR+I+G4 substitution model selected by RAxML-NG v1.1 under the Bayesian Information Criterion (BIC). Branch support was assessed with 1,000 standard bootstrap replicates. BI analysis was performed in MrBayes v3.2.7a (Ronquist et al. 2012) under the SYM+G model. Two independent runs of 10,000,000 generations each were executed, sampling trees every 1,000 generations after a 25% burn-in. Four Markov chain Monte Carlo (MCMC) chains were employed, starting from random trees.

## ﻿Results and discussion

### ﻿Morphological comparison

Through examination of type specimen descriptions and high-resolution images from JSTOR Global Plants (http://plants.jstor.org), *Dysosma xishuiensis* is morphologically distinguished from congeners by the following diagnostic characters. *D. xishuiensis* shares similarities with *D. versipellis* in leaf shape, phyllotaxy, and floral attachment position. However, it is readily distinguishable by the following characters: glabrous abaxial leaf surfaces, pedicels, and sepals (vs. pubescent); 8–20-flowered inflorescences (vs. 5–8); petals obovate–oblong (vs. spatulate–obovate) and larger (2.8–3.4 × 1.5–2.0 cm vs. 2.5 × 0.8 cm); and an obpyriform ovary (vs. ellipsoid). Compared to *D. majoensis*, it is glabrous throughout (vs. puberulent on stems, abaxial leaf surfaces, and pedicels), with uniformly green leaf surfaces (vs. dark green adaxial and grey–purple abaxial surfaces) and obovate–oblong petals (vs. elliptic–lanceolate). Relative to *D. pleiantha*, it displays alternate leaves (vs. opposite), deeply divided leaves (vs. lobed), and flowers attached near the base of the blade (vs. attached near the base of the petiole) (Table 2).

**Table 2.** Morphological comparison of *Dysosma xishuiensis* with four congeners. Data of the four *Dysosma* species are sourced from the “Flora of China”.

Characters	*D. xishuiensis*	*D. majoensis*	*D. pleiantha*	*D. versipellis*	*D. difformis*
**Plant height**	80–120 cm tall	ca. 50 cm tall	20–60 (–80) cm tall	40–150 cm tall	15–30 cm tall
**Leaf blade**	glabrous; palmately 5–8 deeply divided	abaxially puberulent, deeply 4–6-divided, lobes 3-fid at apex	glabrous; 5–9-lobed	abaxially pubescent; palmately 4–9-lobed	glabrous; entire or lobed
**Stem and Petiole**	glabrous	puberulent	glabrous	glabrous	glabrous
**Inflorescence**	attached near base of blade; 8–20-fascicled flowers	attached near base of blade; 2–5 flowers	attached near base of petiole; 5–8-fascicled flowers	attached near base of blade, 5–8-fascicled flowers	attached near base of blade. 2–5-fascicled flowers
**Pedicel**	ca. 5 cm, glabrous	1–3 cm, long puberulent	2–4 cm, glabrous	length unknown, with pubescent	1–2 cm, sparsely white pubescen
**Sepals**	long elliptic, 1.5–2.0 cm × 5.0–8.0 mm, glabrous	elliptic, 7–15 mm, glabrous	elliptic-oblong or ovate-oblong, 1–2 × ca. 0.8 cm	oblong-elliptic, 0.6–1.8 cm × 3.0–8.0 mm, outside puberulent	oblong-lanceolate, 2–2.5 cm × 2–5 mm, outside pubescent, inside glabrous
**Petal**	obovate-oblong, 2.8–3.4 × 1.5–2.0 cm	elliptic-lanceolate, ca. 9 × 1.5 cm	obovate-oblong, 3–4 × 1–1.3 cm	spatulate-obovate, 2.5 × 0.8 cm	oblong-loriform, 4–5 × 0.8–1 cm
**Pistil**	ovary obpyriform, ca.1.5 cm long; style ca. 3 mm long, stigma shield-shaped	Ovary oblong; stigmas shield-shaped	ovary oblong, ca. 1.3 cm; style ca. 3 mm	ovary ellipsoid, glabrous; style short; stigma shield-shaped	ovary urceolate; ca. 0.9 cm; style ca. 2 mm; stigma shield-shaped

### ﻿Phylogenetic relationships

**Figure 1.**
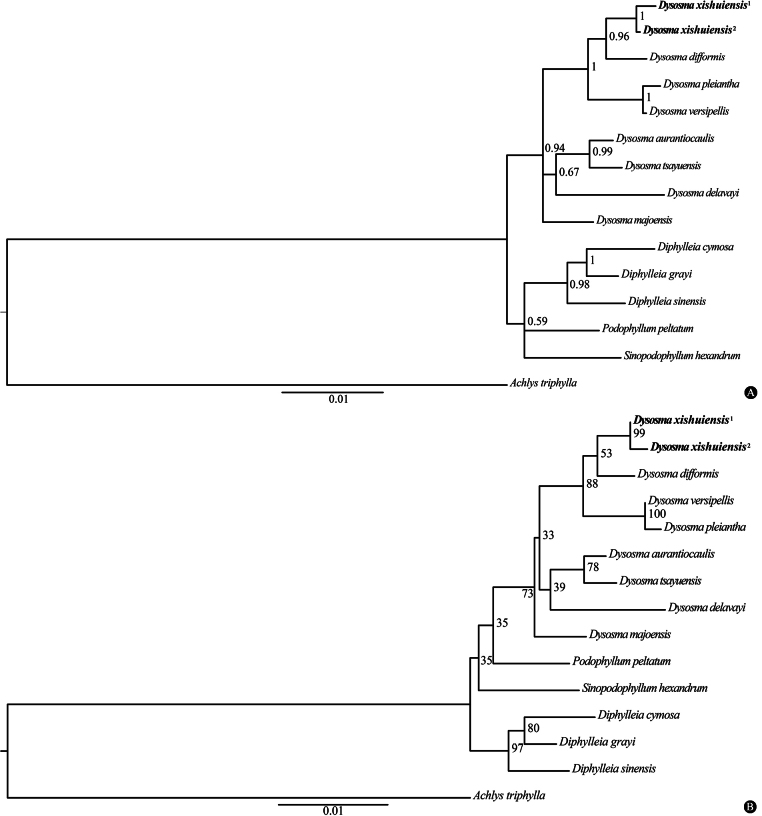
The Bayesian inference phylogeny of *Dysosma xishuiensis* and its close relatives based on the nuclear DNA (ITS) and plastid gene regions (*matK* and *rbcL*). Bayesian inference posterior probability (PP ≥ 0.5; Fig. 1A) and maximum likelihood bootstrap percentages (BP ≥ 50%; Fig. 1B) are shown above the main branches. The samples of the new species are displayed in bold, with superscripts designating two distinct populations.

The combined ITS, *matK*, and *rbcL* datasets yielded a concatenated alignment of 2,051 bp. The phylogeny placed *Dysosma xishuiensis* in a strongly supported clade (PP = 1.00, BP = 99) with other *Dysosma* species (Fig. 1A, B), while its two geographically isolated populations comprised a monophyletic lineage, affirming taxonomic distinctiveness. It is noteworthy that the phylogenetic analysis revealed *D. xishuiensis* and *D. difformis* formed a sister-group relationship, collectively constituting a strongly supported clade (PP = 1.00, BP = 88) with *D. pleiantha* and *D. versipellis*. Morphologically, *D. xishuiensis* differs from *D. pleiantha* and *D. versipellis* as previously described and from *D. difformis* in leaf shape (suborbicular vs. obliquely peltate), pedicels (glabrous vs. sparsely white pubescent), and sepals (glabrous vs. pubescent externally).

### ﻿Taxonomic treatment

#### ﻿ Dysosma xishuiensis

Taxon classificationPlantaeRanunculalesBerberidaceae

Y. B. Yang, M. T. An & C. H. Yang sp. nov.

0DB10667-1F51-527B-AF8E-00062746F2BA

urn:lsid:ipni.org:names:77373457-1

Fig. 2

##### Diagnosis.

*Dysosma xishuiensis* is morphologically most similar to *D. versipellis* in its stems (erect, unbranched, glabrous), leaves (alternate, blade suborbicular, to 25–30 cm in diam, palmately lobed), flowers (attached near base of blade, red), but differs by the following characters: leaf abaxial surface, pedicels, and sepals glabrous (vs. pubescent); inflorescences bearing more flowers (8–20 vs. 5–8); larger obovate-oblong petals (2.8–3.4 × 1.5–2.0 cm vs. 2.5 × 0.8 cm); and ovary obpyriform (vs. ellipsoid).

##### Type.

China • Guizhou, Xishui County, Sanchahe Town, Xishui National Nature Reserve, alt. 975 m, under broadleaf forest beside the stream of Danxia landform, 20 May 2022, *Yan-Bing Yang, Cheng-Hua Yang & He Li* (holotype: GF!; isotype: GZAC!). • Xishui County, Tucheng Town, Tongyi Village, Xishui National Nature Reserve, alt. 1168 m, in the middle of a mountain under broadleaf forest of Danxia landform, 22 May 2022, Cheng-Hua Yang, He Li & Mao Li C1031 (isotype: KUN!).

##### Description.

Herbs, 80–120 cm tall. Rhizomes stout, densely fibrous. Aerial stems erect, pale green, unbranched, glabrous. Leaves alternate; petioles of lower leaves 10–20 cm, upper leaves ca. 3 cm; leaf blade suborbicular, up to 25 cm in diameter, thinly papery, glabrous on both surfaces, abaxially with prominent venation, palmately 5–8-lobed, lobes deeply divided (ca. 2/3 of radius); lobes obovate to obovate-oblong, margins remotely serrate, apex shallowly 3-lobed or bearing 2–3 mucronate teeth. Inflorescence an 8–20-flowered fascicle. Pedicels pendulous, slender, glabrous. Flowers dark red, attached near leaf base. Sepals long-elliptic, 1.5–2.0 × 0.5–0.8 cm, glabrous, apex acute. Petals oblong-obovate, 2.8–3.4 × 1.5–2.0 cm, glabrous. Stamens ca. 2 cm; filaments shorter than anthers; anther connective slightly prolonged, glabrous, acute. Pistil ca. 1.5 cm; ovary obpyriform, glabrous; style ca. 0.3 cm; stigma shield-shaped. Berry ellipsoid. Seeds numerous.

**Figure 2.**
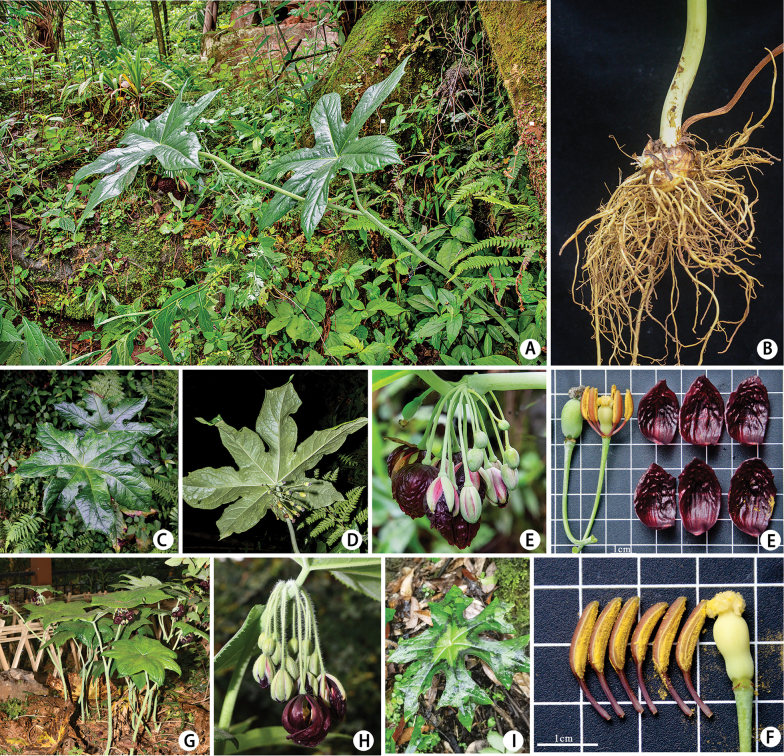
Images of living plants of *Dysosma xishuiensis* Y. B. Yang, M. T. An & C. H. Yang. **A.** Individual; **B.** Root; **C.** An adaxially leaf blade; **D.** Abaxial leaf blade; **E.** Inflorescence anatomy of a flower; **F.** Stamens and pistils; **G.** Plant habit and leaf morphology (*D. versipellis*); **H.** Inflorescence (*D. versipellis*); **I.** Leaf morphology (*D. majoensis*). Photos credit: **A, F, G, I** by C. H. Yang; **C, D** by L. Huang; **B, E** by J. G. Wang; **G, H** by Z. Wei.

##### Phenology.

Flowering from April to June, fruiting from June to September.

##### Distribution and habitat.

*Dysosma xishuiensis* is only known from two localities in Xishui County, Guizhou, China, where it grows under the broadleaf forest by the stream or in the middle of a mountain of Danxia landform.

##### Preliminary conservation status.

Currently, *Dysosma xishuiensis* is documented exclusively in two discrete populations within Xishui National Nature Reserve. These populations occur in mid-subtropical to warm temperate zones characterized by a humid monsoon climate. The species exhibits a restricted distribution range, with both populations each comprising fewer than 100 mature individuals. In accordance with the International Union for Conservation of Nature (IUCN) Red List criteria B2 (a, b(iii)) (extent of occurrence < 500 km^2^, fewer than five locations, continuing decline in habitat quality) and D (population size < 250 mature individuals), we propose that *D. xishuiensis* be designated as Endangered (EN) (IUCN 2022).

##### Etymology.

The species epithet “*xishuiensis*” refers to the type locality of the new species.

##### Vernacular name.

The Chinese name is “xí shuĬ bā jiăo lián” (习水八角莲).

## Supplementary Material

XML Treatment for Dysosma xishuiensis

## ﻿Additional information

### Use of AI

No use of AI was reported.

### Author ORCIDs

Lang Huang https://orcid.org/0009-0004-8038-7432

Wei-Hao Yao https://orcid.org/0000-0002-5941-676X

Yan-Bing Yang https://orcid.org/0009-0007-9014-1817

Ming-Tai An https://orcid.org/0000-0003-3886-0287

Yuan-Lin Yu https://orcid.org/0009-0005-5875-511X

Mei Zhou https://orcid.org/0009-0000-3806-1333

He Li https://orcid.org/0009-0006-0597-3209

### Data availability

The newly obtained ITS, *matK*, and *rbcL* sequences have been submitted to NCBI. All other data that support the findings of this study are available in the main text.
